# From beat tracking to beat expectation: Cognitive-based beat tracking for capturing pulse clarity through time

**DOI:** 10.1371/journal.pone.0242207

**Published:** 2020-11-18

**Authors:** Martin Alejandro Miguel, Mariano Sigman, Diego Fernandez Slezak

**Affiliations:** 1 Laboratorio de Inteligencia Artificial Aplicada, Departamento de Computación, Universidad de Buenos Aires, Buenos Aires, Argentina; 2 Instituto de Investigación en Ciencias de la Computación (ICC), CONICET-Universidad de Buenos Aires, Buenos Aires, Argentina; 3 Laboratorio de Neurociencia, Universidad Torcuato Di Tella, Buenos Aires, Argentina; 4 Consejo Nacional de Investigaciones Centóficas y Técnicas (CONICET), Buenos Aires, Argentina; 5 Facultad de Lenguas y Educación, Universidad Nebrija, Madrid, Spain; IBM Thomas J Watson Research Center, UNITED STATES

## Abstract

Pulse is the base timing to which western music is commonly notated, generally expressed by a listener by performing periodic taps with their hand or foot. This cognitive construction helps organize the perception of timed events in music and is the most basic expectation in rhythms. The analysis of expectations, and more specifically the strength with which the beat is felt—the pulse clarity—has been used to analyze affect in music. Most computational models of pulse clarity, and rhythmic expectation in general, analyze the input as a whole, without exhibiting changes through a rhythmic passage. We present Tactus Hypothesis Tracker (THT), a model of pulse clarity over time intended for symbolic rhythmic stimuli. The model was developed based on ideas of beat tracking models that extract beat times from musical stimuli. Our model also produces possible beat interpretations for the rhythm, a fitness score for each interpretation and how these evolve in time. We evaluated the model’s pulse clarity by contrasting against tapping variability of human annotators achieving results comparable to a state-of-the-art pulse clarity model. We also analyzed the clarity metric dynamics on synthetic data that introduced changes in the beat, showing that our model presented doubt in the pulse estimation process and adapted accordingly to beat changes. Finally, we assessed if the beat tracking generated by the model was correct regarding listeners tapping data. We compared our beat tracking results with previous beat tracking models. The THT model beat tracking output showed generally correct estimations in phase but exhibits a bias towards a musically correct subdivision of the beat.

## Introduction

Music is among life greatest pleasures. Most prominent theories about how music generates affect center on expectations. Music contains repetitions that allow creating abstractions of its structure and predicting how it will evolve. An initial proposal centering on musical expectations was given by Meyer [1]. He claimed that unfulfilled expectations engaged us to take action when events developed differently than planned. Failed expectations invoke an affective response. Later, this theory was further developed by Huron [2]. He proposed that our ability to make predictions about our world was a biological advantage and therefore correct predictions should be rewarded. Music then became a pleasurable activity as it engaged our prediction reward mechanisms by their repetitive nature. Most recently, Vuust and Witek [3] reframed these ideas under the Predictive Coding framework of cognition. Predictive Coding suggests the brain is a prediction machine that attempts to reduce prediction errors. Predictions are performed in a hierarchical fashion, from more abstract representations that condition less abstract predictions. Updates on the model occur when predictions fail. The authors propose the backpropagation of the prediction error is causal to the affective response.

Empirical studies have studied how expectations over the distribution of musical events in time, its rhythm, relate to affect. Two main features related to expectations have been commonly used in this exploration. One of them is the strength of the feeling of a beat, often referred to as *pulse clarity*. The beat, pulse or *tactus* of a song is commonly expressed by a listener by tapping a foot or hand in synchrony to it. It constitutes a commonly isochronous pulse that is used to describe the location in time of musical events and is the most basic rhythmic expectation. The ability to extract such regularity from musical stimuli is almost exclusive to humans and plays a decisive role in our understanding of music [4]. In psychological experiments, *pulse clarity* metrics of the stimuli are generally obtained using the *MIRToolbox* [5], a Matlab toolbox that contains algorithms for music information retrieval. The toolbox allows extracting a pulse clarity metric from auditory musical stimuli [6]. This feature has been shown to be related with the strength and precision of people’s movements to music [7, 8] and to the perceived valence of classical music excerpts [9]. Another relevant metric that revolves around expectations has been *rhythmic complexity*, a concept referring to how summarizable a rhythm is. Rhythmic complexity has been used to analyze affect in symbolic rhythmic stimuli [10]. These models only provide summary metrics for short excerpts where rhythmic onsets are set precisely on the beat grid [11]. They often require a prior definition of the beat of the stimulus and cannot be used on rhythm with microtiming variations such as those found in real performances.

Rhythmic expectations are generated, fulfilled or diverted as a musical passage unfolds. The analysis of expectation and affect can be enhanced by looking into its dynamics in time. To the best of our knowledge, there are no models to analyze these expectations over time on symbolic stimuli. In this work we address the gap in models by presenting the Tactus Hypothesis Tracker model (THT for short), a model of pulse clarity over time for symbolic input. The model proposed has the following features:

- Analyses possible beat interpretations for a rhythmic passage.
- Provides an ongoing metric of the congruency of the beat interpretation, which will be considered a metric of *pulse clarity*.
- Works causally, that is, performs estimations using only information previous to the point of estimation. It does not use future information.
- It is intended to work on symbolic data, more specifically onset times.
- It works on examples where the time between beat times (inter-beat-interval or ibi) is not necessarily constant and the interval between musical onsets (inter-onset-interval or ioi) are not necessarily multiples of a base duration. This requires the model to adapt to microtiming variations. This input is often named as *expressive*.

We focus on the beat as it is a cognitive percept required for the understanding, synchronization and communication of the musical experience [4]. We center on symbolic data for it to be useful in experiments focusing on the timing of rhythmic events in designed stimuli. We also seek for the model to provide estimations of which is the underlying beat, together with its clarity, so they can be used to inform models capturing more abstract rhythmic constructs based on the beat (e.g.: the meter).

We propose to look into models of beat tracking as the basis of our proposed model. Beat tracking is the task of defining the points in time where a human listener would tap the beat if listening to a song. The task originates from the field of Music Information Retrieval, where algorithms are designed to obtain high level features from an audio signal or music in symbolic format. Beat tracking is useful to inform other tasks such as chord change detection, chord identification, cover song detection or tempo estimation [12].

A great variety of algorithms and techniques have been proposed for the problem. First attempts used rule-based models. These rules attempted to mimic a person’s listening algorithm [13, 14] or find repeating patterns [15]. Next, agent-based algorithms were proposed, where each agent conceiving one possible interpretation of the rhythm listens to the musical passage. The agents are finally scored to choose a winning interpretation [16, 17]. On a similar venue, some proposals explored the hypothesis space taking advantage of dynamic programming techniques, which enhances the exploration process [18–20]. [21] is a probabilistic beat tracking algorithm using Kalman Filters on an activation function obtained from onset times from symbolic data. [22] presents a distinct envision of the process, where two populations of non-linear oscillators with different natural frequencies are coupled. These populations reflect the connections between motor and auditory areas in the brain. Finally, most recent approaches rely heavily on neural networks, either convolutional [12] or recurrent [23].

Model’s input can be either symbolic (MIDI or other representation of musical events) or an audio signal. In most cases, audio signals are pre-processed to obtain either onset times [17] or an onset detection function describing the likelihood of finding an onset at a given time [24]. Neural network approaches may skip the onset detection phase and estimate a beat activation function instead. This function defines the likelihood of finding a beat at a given time. The beat function is then filtered to obtain discrete beat times. Most of these algorithms analyze the entire signal to estimate the initial parameters of the beat tracking process, which is then done in a causal fashion (from beginning to end without using future information). Some approaches work entirely causally [12] or have been adapted to do so [25, 26].

Efforts in the beat tracking task are gathered yearly in the Music Information Retrieval Evaluaton eXchange (MIREX). The exchange hosts a variety of MIR competitions, including beat tracking. A particularly salient model in the last few years is the one by Böck et al. 2016 [23].

Our proposal is a beat tracking model that continuously produces a pulse clarity metric and can be used for experiments on the relationship of rhythmic expectations with affect. With this in mind, we aim for a simple model that allows understanding how pulse clarity is calculated from the stimulus.

We model rhythms as simple sequences of events in time. Despite this simplification, there is evidence that even in simple rhythmic patterns expectations emerge. Dehaene, Meyniel, Wacongne, Wang and Pallier [27] and Honing, Ladinig, Háden and Winkler [28] have shown that missing or changed events in such circumstances create physiological responses. We also require the model to be entirely causal, given that expectations are created as we listen to a song. Finally, we refrain from modeling prior knowledge of music style. Although such priors are relevant, evidence has shown that expectations emerge even without them, for example in newborn babies [28] or in music from foreign cultures [29].

Our proposed model is based on the agent-based models from Dixon [17] and Rosenthal [16] as they work on symbolic input, are fairly transparent and can be easily adapted to perform causally.

In the next section we describe the **Tactus Hypothesis Tracker** model. The **Evaluation** section presents our evaluation of the model. We evaluated its *pulse clarity* metric comparing it against human beat tapping variability. We used the training dataset from the MIREX Beat Tracking competition [30]. Our results were compared against the pulse clarity metric from the MIRToolbox [5]. Next, to analyze how the pulse clarity metric behaves in time, we designed four synthetic rhythms that present situations where the beat changes or is less overt and see whether the clarity is affected by the changes. Finally, we assessed whether the beat tracking produced by the model is correct. We did so over the MIREX dataset. We compared the results against the performance of Dixon’s BeatRoot model [17], as well as the state-of-the-art models from Böck et al. 2016 and 2017 [23, 25]. The **Discussion** revises the results in the broader context of analysis of musical affect and proposes further developments for the model and its uses as a tool for experimental designs.

## 1 Tactus hypothesis tracker system

This work presents the *Tactus Hypothesis Tracker* model (THT for short), a beat tracking model intended to produce a pulse clarity metric over time. The model is based on previous efforts on the beat tracking task and has adaptations that make its inner workings part of the output. The adaptations allow inspecting whether several beat hypotheses are prominent, how well they represent the beat and whether this changes over time. The objective is to provide a tool for exploring changes of pulse clarity in symbolic stimuli.

THT is an agent-based model, where each agent represents one possible beat tracking, a *tactus hypothesis*. Each hypothesis is developed as the rhythmic passage unfolds. Throughout the process, each hypothesis is given a *congruency score* reflecting how fit its beat tracking is for the rhythmic passage. This modeling can represent different pulse clarity situations. For example, if a rhythmic passage has a very obvious pulse, one hypothesis should stand out with a very high score. If the passage could be understood under different tacti, the model would have several distinct hypotheses competing with each other. A passage could induce only one pulse but very weakly. In this case, only one hypothesis would stand out but would also score poorly. A passage may also induce different beats in different sections. The model should express this with changes in the scores of the beat hypotheses over time. The THT model takes as an input a sequence (*r*_*i*_) of the onset times of the rhythmic events. As an output, it provides the entire evolution of each tactus hypothesis tracked.

**Fig 1:**
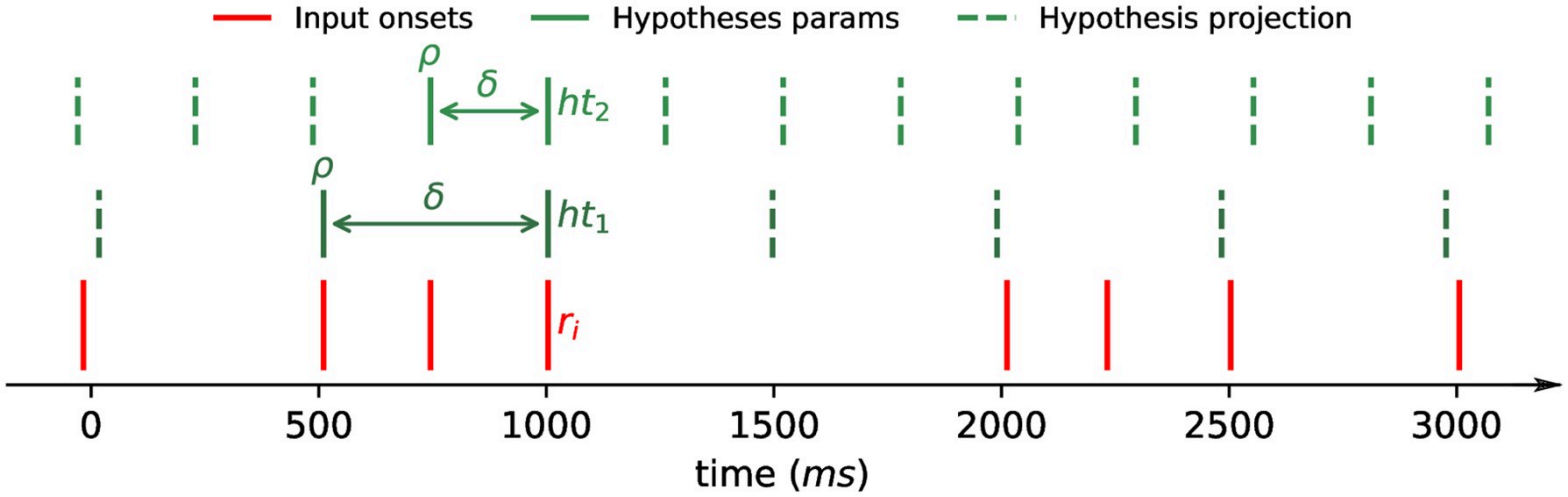
Depiction of an input (*r*_*i*_) and two tactus hypotheses (*ht*_1_ and *ht*_2_), their parameters and projections.

We consider a possible tactus as an isochronous pulse, as the one produced by a pacemaker. The pulse is described by a period (*δ*) and a phase (*ρ*). The period is the time interval between beat times, starting from the time established by the phase. We name the beat times generated from these two parameters the projection of the tactus hypothesis ($p_{k}^{\rho ,\delta }$ or $p_{k}^{h}$). Given a tactus hypothesis, a projection is described by the sequence in Eq 1. The projection is limited as to not extend beyond the rhythmic passage in either direction. Two hypotheses, their projections and an input sequence are depicted in Fig 1.

$$\begin{array}{c} p_{k}^{\rho ,\delta }=\rho +k\times \delta \;\text{with}\;k\in Z \end{array}$$ (1)

**Equation 1**. Hypothesis projection definition.

**Fig 2:**
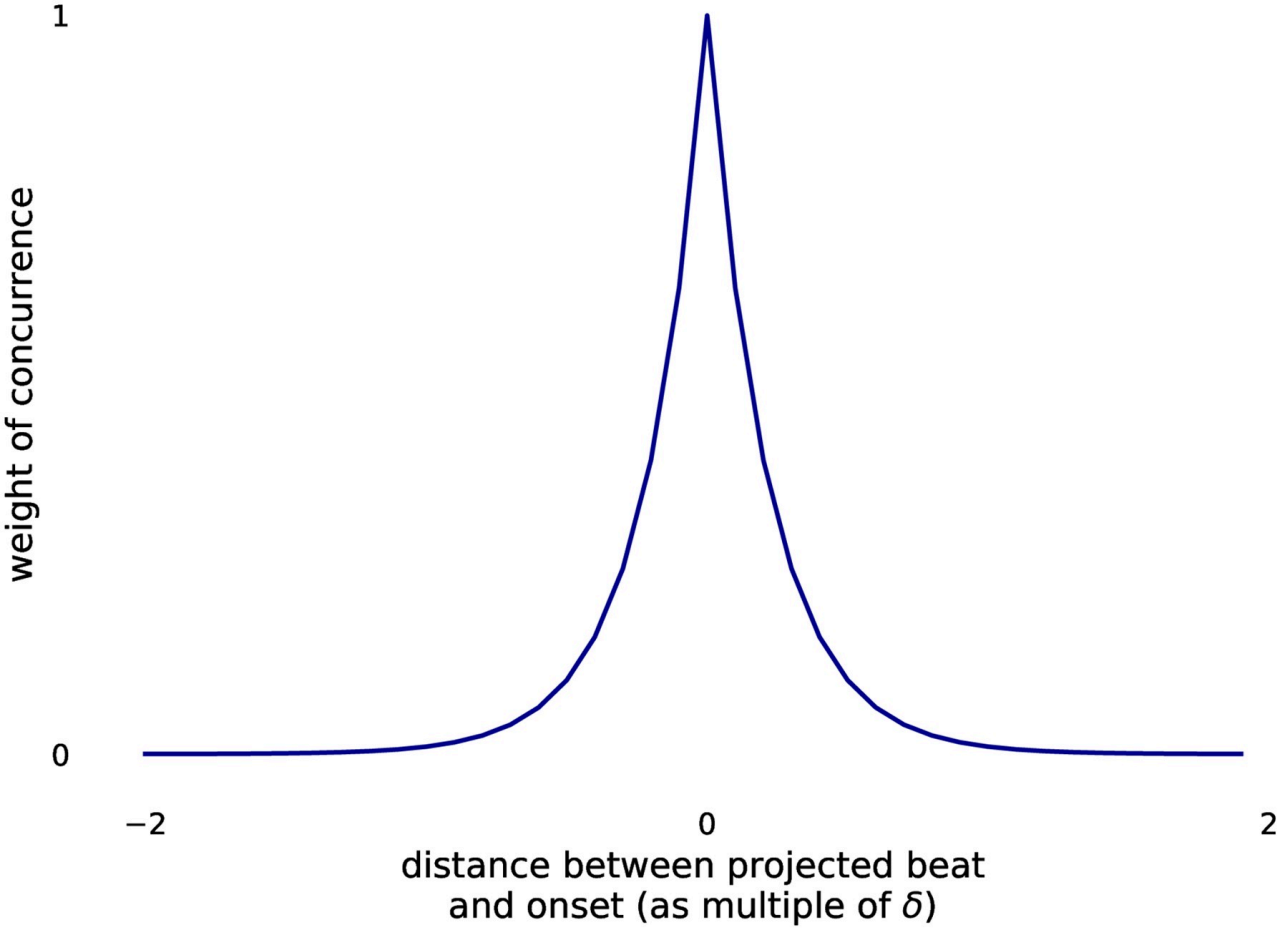
Shape of the concurrence weight function.

Given a rhythmic input (*r*_*i*_), each hypothesis is assigned a *congruency score* that measures its fitness to the input. The score should indicate how suitable it is as the beat felt by a listener. Evidence regarding when a tactus hypothesis is good for a listener comes from [31]. In their work, they argue and then tested that feeling the beat is key to understanding music and a good tactus for this aim is one that coincides with most musical events. A good tactus hypothesis should also have few beat times without rhythmic events. We designed our score to be bounded between 0 and 1, being 1 the case where the tactus is very likely to be the one tracked by the listener. We score the congruency of a hypothesis as the product of the precision by the sensitivity of the beat projection—that is, how many projected beat times are actual rhythmic onsets and how many rhythmic onsets are designated as beat times. To calculate the score, each projected beat time ($p_{k}^{h}$) is matched with the closest input’s onset time, which we named *r*_*k*_. Whether a beat time concurs with an event is weighted by the distance between them. The final equation for the score is given in Eq 2 and the equation that weights the concurrence of events is given in Eq 3. The shape of the concurrence function is depicted in Fig 2. The congruency score is parametrized according to how much context of the passage before the last onset heard should be used. The time window was set to 6 seconds.

$$\begin{array}{cc} congruency(h,\left(r_{i}\right))= & \frac{\text{hits}\;\text{by}\;\text{hypothesis}}{\text{predictions}\;\text{by}\;\text{hypothesis}}\times \frac{\text{hits}\;\text{by}\;\text{hypothesis}}{\text{music}\;\text{events}}\\ \text{hits}\;\text{by}\;\text{hypothesis}= & \sum_{k}concurrence\left(p_{k}^{h},r_{k}\right)\\ \text{predictions}\;\text{by}\;\text{hypothesis}= & \left|(p_{k}^{h})\right|\\ \text{music}\;\text{events}= & \left|(r_{i})\right| \end{array}$$ (2)

**Equation 2**. Congruency score.

$$\begin{array}{c} concurrence\left(p_{k},r_{k}\right)=0.01^{\frac{|p_{k}-r_{k}|}{\delta_{h}}} \end{array}$$ (3)

**Equation 3**. Weight of concurrence distance.

**Fig 3:**
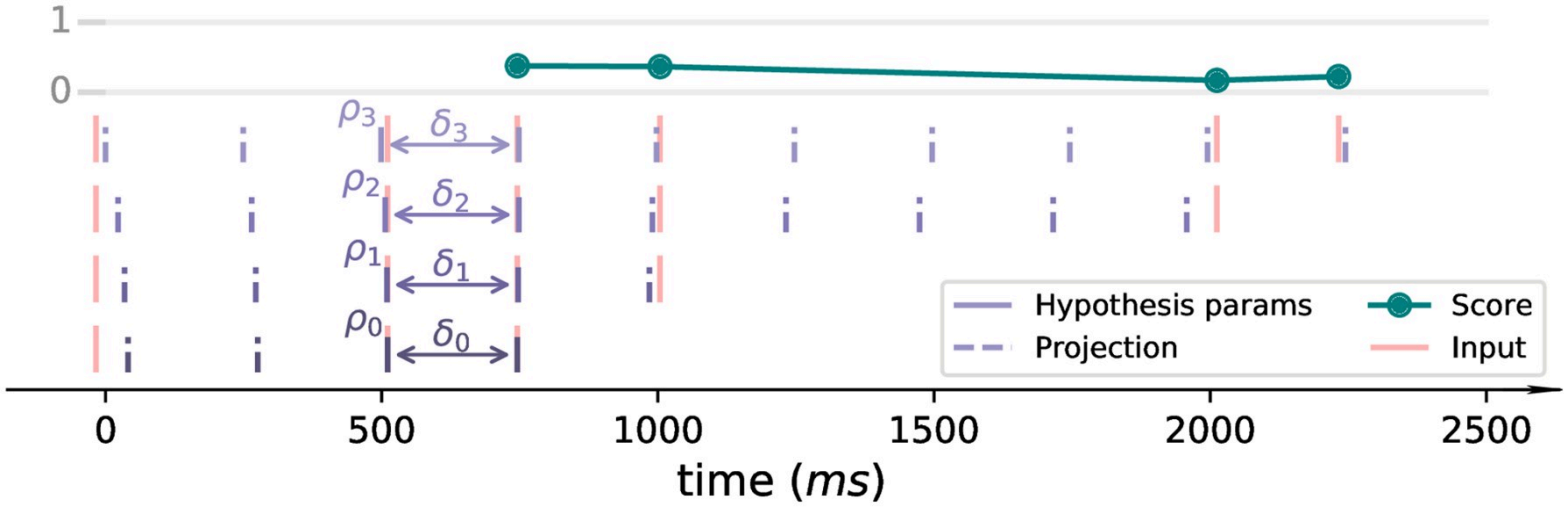
Depiction of a hypothesis tracker and its evolution. First four rows (bottom to top) present the history of the hypothesis tracker parameters (*ρ*_1..4_ and *δ*_1..4_) after processing four new onsets. Top row presents the hypothesis congruency score values after each new onset is ‘heard’.

The THT model works by performing the beat tracking procedure while ‘listening’ to the rhythmic passage. Each onset is processed sequentially updating the state of the agents. With each new onset, new tactus hypotheses are created, hypothesis parameters of existing trackers are corrected to fit the passage better, and each tracker’s score is updated considering the extended passage. Hypothesis trackers keep track of their entire evolution, that is, the parameters and congruency score values in each onset since created. Fig 3 depicts a hypothesis tracker and how its parameters and score changes after each new onset is encountered.

**Fig 4:**
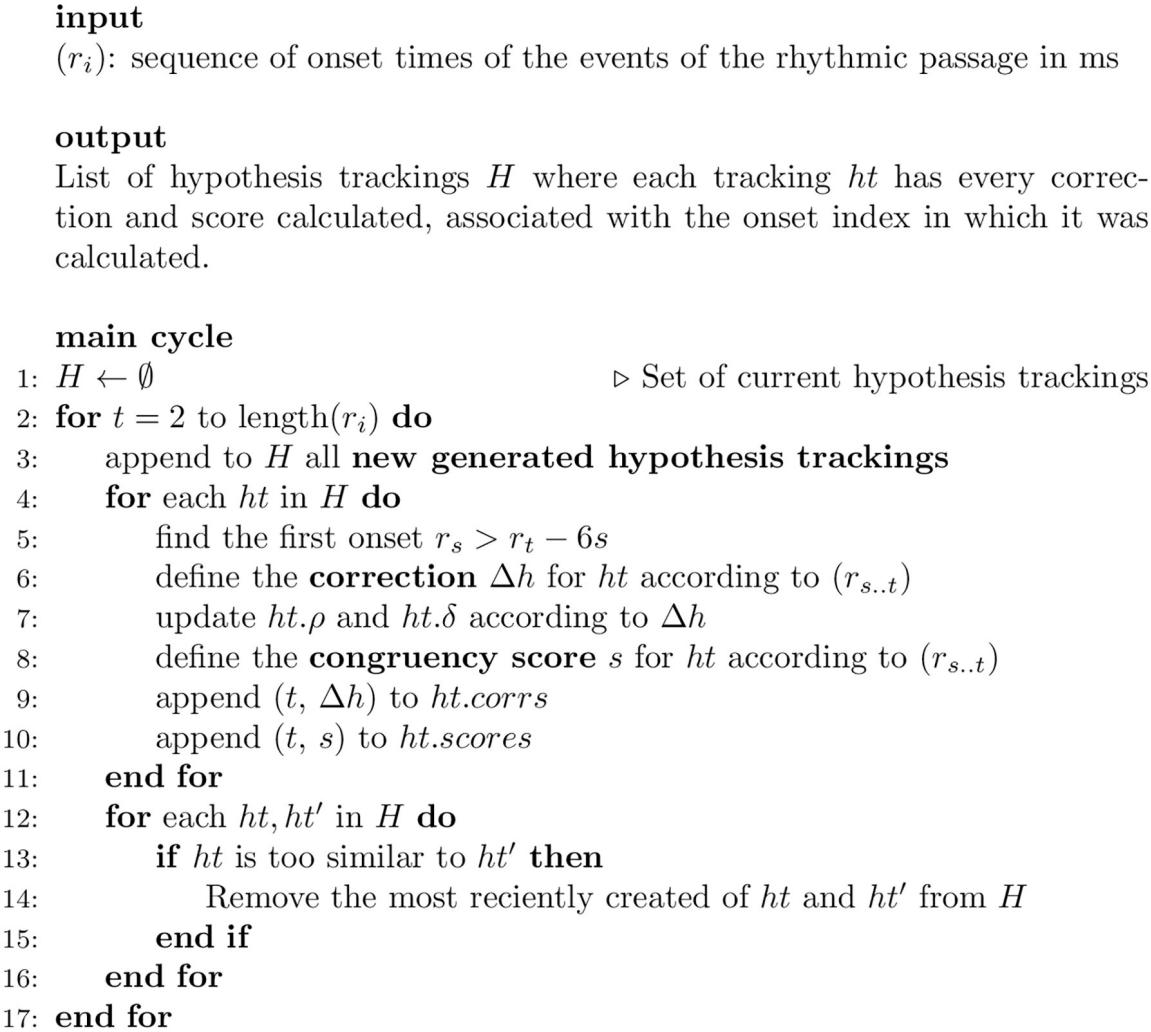
Description of the model’s main loop.

Hypotheses with similar period and same relative phase will be tracking the same beat. To manage this, after processing a new onset, trackers that are too similar to each other are collapsed into one by preserving the oldest one. This processing loop is depicted in more detail in pseudocode in Fig 4. How hypotheses are generated and corrected is described in detail in the next subsections.

As output, THT produces either a binary representation of the *hypothesis trackers* or a human-readable table. An example table output is presented in Table 1. The rows of the resulting table describe the state of a hypothesis tracker after being updated on a given onset event. Each tracker is defined by the initial tactus hypothesis parameters. Hypotheses are created from onset times (see next subsection) and are identified by the index of the onsets used (columns *a* and *b*). The state is described by current *ρ* and *δ* values as well as the congruency score. This output is further processed to obtain a beat tracking and pulse clarity profile over time by selecting the highest scoring hypothesis at each timestep (see **Evaluation** section for further details).

**Table 1:** Example rows of the table output of the THT model.

a	b	onset_index	onset_time	period	phase	score
0	1	1	501.042	500	1.04167	1
0	1	2	1001.04	500	1.04167	1
0	1	3	1501.04	500	1.04167	1
			⋯			
0	2	2	1001.04	1000	1.04167	0.666667
0	2	3	1501.04	1000	1.04167	0.5
0	2	4	2001.04	1000	1.04167	0.6
			⋯			
8	9	9	4751.04	499.045	4248.59	0.074675
8	9	10	5251.04	498.88	4251.8	0.116558
8	9	11	5751.04	498.44	4254.02	0.155319
			⋯			

Each row indicates a hypothesis tracker state at a given time. *a* and *b* columns indicate the onset indexes used to generate the hypothesis tracker; they identify the tracker. *onset_index* indicates the onset index of the tracker state in the row. *onset_time* is the time in milliseconds corresponding to that onset index. *score*, *phase* and *period* are the tracker’s score and parameters at the given time.

The code implementing the model is available at [32]. See the **Data and software availability** section for further information.

### 1.1 Generating new hypotheses

To define a hypothesis we need a phase and a period. We will consider as possible parameter values any period found between two onsets in the input using the first onset as the phase. That is, given two onset times *r*_*a*_ and *r*_*b*_ with *a* < *b*, we have a possible hypothesis with phase *ρ* = *r*_*a*_ and period *δ* = *r*_*b*_ − *r*_*a*_. The set of hypotheses is extended with each newly discovered onset (see line 3 in Fig 4). During this step, the last onset heard, *r*_*t*_, is considered as *r*_*b*_ and a previously heard onset is used as *r*_*a*_. We only consider previous onsets where the period *r*_*b*_ − *r*_*a*_ is between 187ms and 1500ms (equivalent to 320 and 40 bpm, respectively).

### 1.2 Hypothesis correction

**Fig 5:**
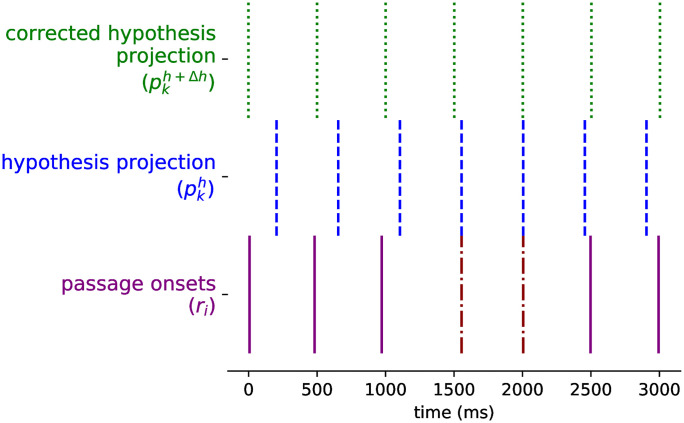
Example of how a hypothesis is corrected on a simple isochronous beat. From bottom to top: (*r*_*i*_) the rhythmic passage events with the onsets originating our tactus hypothesis marked differently, $\left(p_{k}^{h}\right)$ the projection of the hypothesis, and $\left(p_{k}^{h+\Delta h}\right)$ the projection of the corrected hypothesis.

The input onset times have timing variations related to expressiveness and error factors. They are not strictly on times that are multiples of a base duration. For this reason, the parameters of the hypotheses will need to be adapted to fit the onsets better. Let us examine the simplest case where the input is an isochronous beat with imperfect timing. Any two consecutive onsets will be a valid hypothesis for the beat, but due to the timing variations, the projected beat will not match the rest of the onsets. In Fig 5, we display the mentioned situation. The hypothesis is created from two contiguous onset times but its projection $\left(p_{k}^{h}\right)$, due to the imperfections on the input times, fails to match the onsets as it extends from its origin.

Because the projection of a hypothesis works as a linear function of its phase and period, we propose to correct these parameters with a linear regression on the *projection error* between the tactus projection times ($p_{k}^{h}$) and their closest onset event (*r*_*k*_). The slope and intercept of the regression will be used as the correction (Δ*h*) of the period and phase of the hypothesis. In Fig 5 we show the example of the correction for a simple isochronous beat. The projection of the corrected hypothesis $\left(p_{k}^{h+\Delta h}\right)$ is a better match for the tactus.

**Fig 6:**
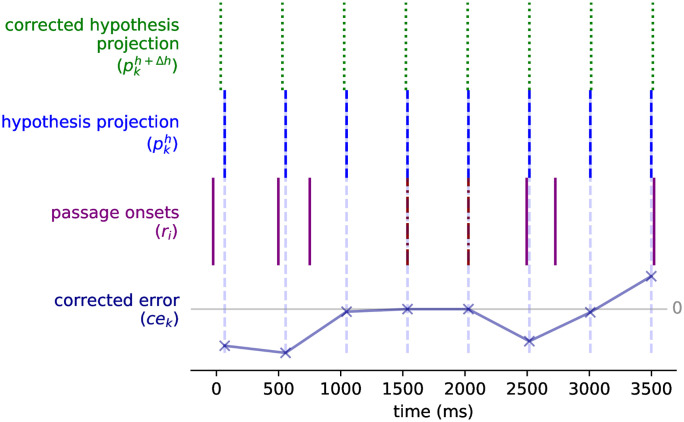
Example of hypothesis correction on a complex rhythm. The figure repeats all three elements from Fig 5: input (*r*_*i*_), original hypothesis projection ($p_{k}^{h}$) and corrected hypothesis projection ($p_{k}^{h+\Delta h}$). The input is now a complex rhythm where not all hypothesis projection times have a corresponding onset time (at 1000 and 3000 ms). The correction is now performed using the corrected prediction error (*ce*_*k*_) for each projection event. The corrected prediction error is shown, at the bottom of the figure. The hypothesis projection lines $\left(p_{k}^{h}\right)$ are extended downwards to match with correction error score. The corrected error is larger when the error is larger but goes back to zero when there is no onset event close to the projection event.

The simple example manages to correct the hypothesis’ parameters since every event of the projection has a corresponding event in the passage. The correction process gets trickier when some tactus events do not have a matching onset. Fig 6 presents an example of this kind of input. Beat times at 1000 and 3000 ms do not have onset times, and non-beat onsets are found at about 1500 and 2750 ms.

We need to detect cases where the tactus projection has no matching input onset time so they are not used in the correction process, otherwise they would add a large erroneous weight to the correction. To accomplish this, we soften the projection error between a projection time $p_{k}^{h}$ and its closest onset *r*_*k*_ proportionally to the distance and relative to the hypothesis’ period *δ*. The expression for the corrected error (*ce*_*k*_) is given in Eq 4. The linear regression is performed over (*ce*_*k*_). The corrected error is 0 when the events are simultaneous, becomes larger with distance from 0 and then converges back to 0. In Eq 4, *m* and *d* are the multiplier and decay parameters, respectively. The *m* parameter defines how sensitive the correction is to the distance between a beat time and its nearest onset time. The *d* parameter adjusts how soon this distance is too large so the error must be ignored. These parameters were set by exploration on a set of artificial examples (*m* = 2, *d* = 0.0001). Another parameter of the correction process is how much context before the current time the model takes into account (see line 6 in Fig 4). Here it was set to 6 seconds.

$$\begin{array}{c} ce_{k}=m\times \left(r_{k}-p_{k}^{h}\right)\times d^{\frac{|p_{k}^{h}-r_{k}|}{\delta }} \end{array}$$ (4)

**Equation 4**. Corrected prediction error definition.

An example of how the corrected projection error is used is presented in Fig 6. After the correction is applied to the projection error, those cases where the tactus has no matching onset have very little error and are therefore mostly ignored by the correction (for example, at times 1000 and 3000 ms). If we compare Figs 5 and 6, the projection of the corrected hypothesis in the first case is closer to the expected beat than in the second case. This is because the correction over the error attenuates the effect of the correction. The problem is solved given that each hypothesis is corrected repeatedly with each new seen event during the beat tracking process (line 6 in Fig 4). This procedure separates relevant timing errors from tactus events that have no matching onset.

## 2 Evaluation

We evaluated the model on three aspects: whether an overall pulse clarity metric obtained from the model corresponds with human perception, whether the clarity metric changes on synthetic rhythmic inputs with changing beat scenarios and whether the final beat tracking performed by the model agrees with beat tracking performed by listeners. On the first and last evaluation, we compared our performance with previous work.

For the first and last evaluations requiring listeners data, we used the training dataset for the MIREX Audio Beat Tracking competition [30]. The dataset consists of 20 song excerpts of 30 seconds each from varying musical genres (e.g.: funk, folk, pop, east european ska and western classical music). Each excerpt was annotated by 40 listeners. The annotation procedure consisted of the listeners tapping to the beat as they found fit into an input device while listening to the song [33].

In the next subsections we present the procedure and results for each peformed evaluation.

### 2.1 Overall pulse clarity

To assess the pulse clarity metric obtained from the THT model, we compared it against the beat tapping data from the MIREX dataset. This dataset was intended to be used in the MIREX beat tracking challenge and was designed to present different rhythmic scenarios by containing a variety of musical genres. THT’s congruency score is designed to reflect how well a tactus matches the rhythmic passage. The higher the score the clearer the beat hypothesis is and a more isochronous tapping would be expected in the annotations. We generated an overall pulse clarity score from the output of the THT model and we inspected the tapping variability of the participants of the dataset.

The overall THT pulse clarity metric was calculated as the mean of the congruency score from the highest scoring hypotheses at each update of the model. During the tracking procedure, each new processed onset involved updating the congruency score of the tactus hypotheses. After each update, we selected the highest scoring hypothesis and kept that score. Finally, we used the mean of these scores as the overall pulse clarity. To apply our model to the MIREX dataset consisting of audio excerpts, the input needed to be converted into onset times. We performed this conversion using the onset detection algorithm from Dixon’s BeatRoot model [17].

To obtain a pulse clarity metric from empirical data, we looked into the annotator’s tapping variability when tapping to the MIREX musical excerpts. We looked into the coefficient of variability (CV) of the inter-tap intervals of each annotator in each track [34]. The normalization of the coefficient of variability considers the variability with respect to the tapping rate selected by the listener. A smaller coefficient indicates a more precise tapping. We first filtered inter-tap interval values larger than 3000ms (indicating a beat slower than 20 bpm). Then we calculated the tapping variability by dividing the standard deviation by the mean of the inter-tap interval distribution. The coefficient of variability (CV) was calculated for each annotator on each track and then an average was obtained for each track.

**Fig 7:**
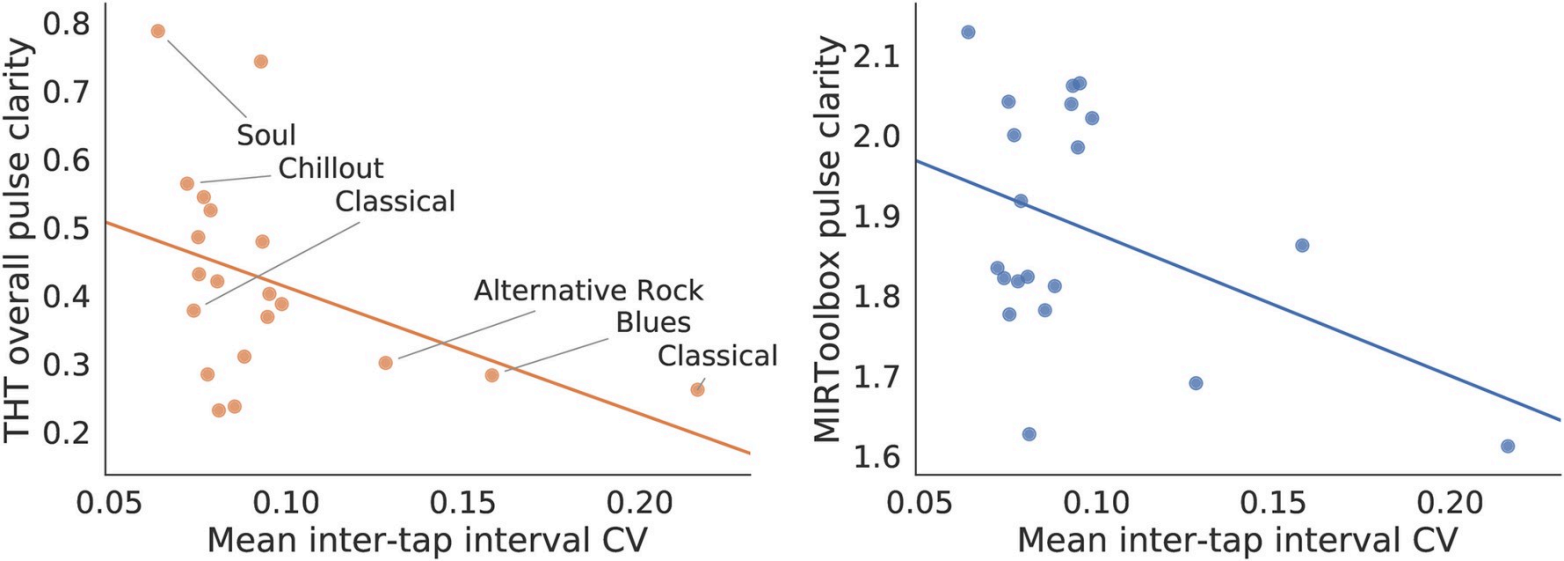
Pulse clarity score compared against tapping variability. Left: THT clarity score is calculated as the mean congruency score of the top hypotheses. Tapping precision is calculated as the mean coefficient of variability of the inter-tap interval distribution. Each point represents one track in the MIREX Beat Tracking training dataset. Spearman correlation has *r* = −0.534 (*p* = 0.015). Genres of least and most variable tapping scenarios (on average) are annotated in the plot. Right: Pulse clarity score from MIRToolbox [5] compared against tapping variability (Spearman *r* = −0.149, *p* = 0.531).

Fig 7 left presents the pulse clarity metric from THT against tapping variability (mean inter-tap interval CV) from the MIREX dataset. For comparison, we also obtained pulse clarity values with the MIRToolbox [5] and presented them against the empirical data (Fig 7, right). A negative correlation was observed between THT’s pulse clarity and tapping precision (Spearman *r* = −0.534, *p* = 0.015). This correlation is greater to the one obtained when contrasting with MIRToolbox (Spearman *r* = −0.149, *p* = 0.531). The negative correlation result shows that the congruency score obtained from the THT model is related with the strength of the beat percept, as expressed by smaller tapping variability associated to higher confidence of the THT model in its beat tracking. The figure also exhibits that the dataset contains a wide range of tapping scenarios, where the greatest inter-tap interval variability is three times larger than the most precise inter-tap interval distribution.

### 2.2 Changes in pulse clarity

**Fig 8:**
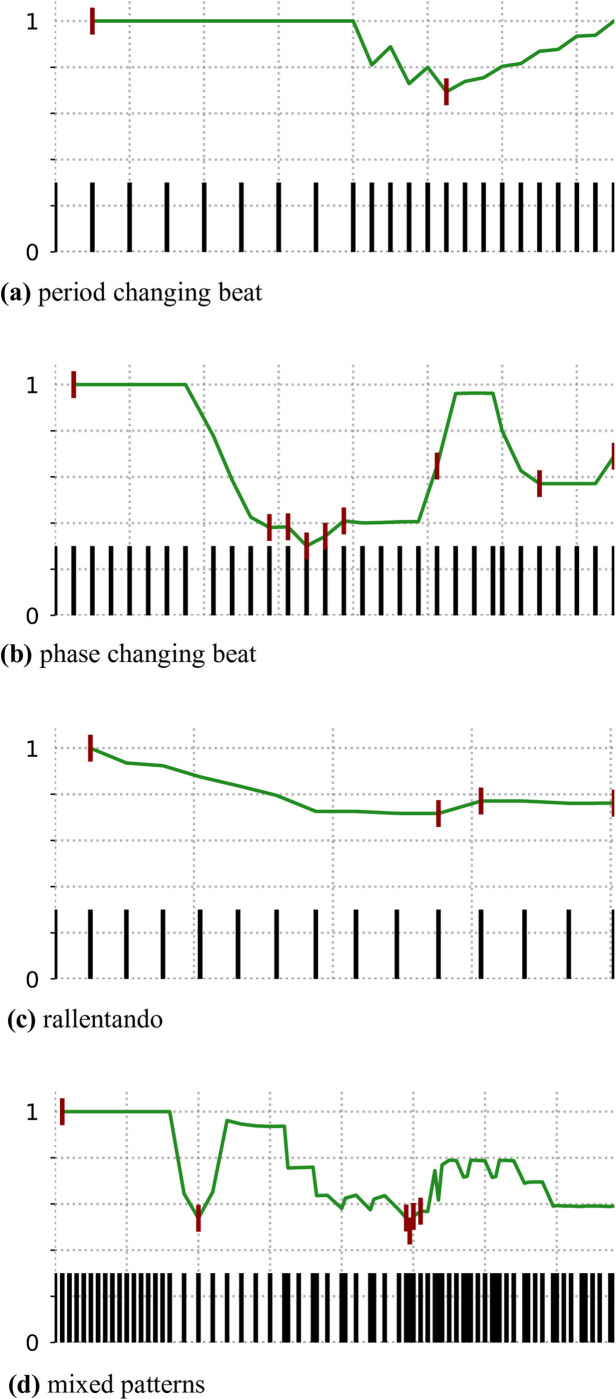
THT’s pulse clarity metric developing over synthetic rhythmic passages. The plots show the confidence of the top hypothesis over time. The X axis is time. The Y axis is the congruency score of the best hypothesis at a given time. Markers are placed each time the main hypothesis changes. At the bottom of the plot, the musical events are displayed over time.

A second evaluation was performed to assess the behaviour of the pulse clarity metric over time. To this end, we proposed four synthetic rhythmic passages in which different behaviors were expected. Because the model does not only provide a pulse clarity metric but information on the entire tracking process, we will also look into whether the top-scoring tactus hypothesis changes. The examples developed for the analysis are depicted in Fig 8 and are as follows:

- **period changing beat**: the passage starts with an isochronous rhythm that becomes twice as fast in the second half.
- **phase changing beat**: the passage starts with an isochronous beat that changes phase twice during the passage. The first change pushes the beat forward half a period. The second change goes back to the first phase.
- **rallentando**: the inter-onset-interval is slightly reduced with each new onset.
- **mixed patterns**: the pattern is composed of 5 different rhythmic subpatterns, some of which are not isochronous pulses.

In the *period changing beat* example we would expect a hypothesis to set with a perfect score in the beginning, given that we are listening to a perfect isochronous rhythm. When the period of the clock changes, this hypothesis should gradually lose congruency until it is overruled by a better hypothesis that matches that period and recovers the score. The *phase changing beat* should produce a similar behavior but with two valleys on the congruency profile, one for each phase change. The *rallentando* example is intended to show another expected feature of the model: the difference between more subtle and more obvious changes in the beat hypothesis. A rallentando is an artistic resource where the song gets progressively slower. It is commonly used at the end of a song. In that case, we would expect the model to slowly adapt the current beat hypothesis to the tempo changes, but it should not change to an entirely different hypothesis. This would represent the case where a listener does not conceive the change in the beat as an entirely new beat. In the last example, *mixed patterns*, we expect the beat hypothesis to be less congruent on average given that the patterns are not simply isochronous onsets. This example should show the sensitivity of the congruency score to varying rhythmic situations.

To analyze the expected behavior of the model to the given examples we plotted the congruency score of the best hypothesis over time and showed when the best hypothesis is overruled by a new one. This demonstrates if and when the hypothesis changes in the first two examples; if the hypothesis does not change in the third and the overall score during the varying passages in the last one. Plots are shown in Fig 8.

We can see the model behaves mostly as expected. In Fig 8a we see how during the isochronous beat there is one perfect hypothesis. Later, after the period change, this hypothesis loses congruency and is soon replaced by a new hypothesis that again is very congruent with the passage. This is very similar to what happens in Fig 8b, once for each phase change. Fig 8c shows the main hypothesis losing congruency with the slow period change, but not being overruled by a new one until three quarters into the passage. Finally, Fig 8d exhibits how different rhythms have different average congruency. In particular, between the second and third and between the fourth and fifth rhythmic pattern, the top tactus hypothesis does not change given that it is still the best option albeit with a low congruency score.

### 2.3 Beat tracking

This subsection evaluates if the highest scoring beat interpretations of the model are consistent with the selected beat of human listeners. This evaluation is not straightforward given that different listeners may select different beat interpretations for the same stimulus [35]. They may tap in counter-phase or at half the period and still produce musically acceptable beat tracks. The MIREX Beat Tracking competition evaluates beat tracking models by comparing the sequence of beat times produced by the model against each beat annotation. To produce a summary, the score comparing the model’s output with each annotation is averaged per song and then among the songlist. In the cases where annotations are very different, the score can be uninformative. We therefore present a comparison of THT’s beat tracking output against models that were designed for the beat tracking task to provide a reference of the quality of the output. We used the MIREX training dataset [30] for the comparison.

We selected three comparison models. We contrasted with Dixon’s BeatRoot [17] (version 0.5.8), an agent-based model with a behavior very similar to THT’s. The main difference is that BeatRoot creates an original set of period values before the beat tracking phase by inspecting the entire musical passage. Additionally, BeatRoot snaps beat times to onset times when possible. In contrast, our model does not select possible period values beforehand. Also, final beat times are generated from the model’s tactus hypotheses and are not fixed to onset times, as not to use future information during the process. Finally, our score function for the agents is bounded between 0 and 1 to be representative of pulse clarity. Both models have tens of parameters which were tuned with synthetic data. They both work iteratively and do not have a restriction on the number of agents that are managed at a given time.

We also compared with the models from Böck et al. 2016 [23] (DBNDownBeatTracker in the *madmom* python package version 0.16.1 [36]) and Böck et al. 2017 [25] (DBNBeatTracker with –online parameter from the same *madmom* package). The model from Böck et al. 2016 [23] is currently one of the highest performing models in the MIREX Beat Tracking competition. It uses an ensemble of Recurrent Neural Networks with Bi-directional Long-Short Term Memory (LSTM) cells adding up to thousands of parameters. They were trained on several hours of beat tracking data to learn a beat activation function. The activation function is then filtered by a Dynamic Bayesian Network to obtain a final beat track. The model from Böck et al. 2017 [25] is an adaptation from of model of 2016 that uses Uni-directional LSTM cells to be able to work online, i.e. it does not use future information.

The comparison seeks to provide an overview of the musical fitness of the beat trackings generated by the THT model. Discussion of model’s complexity is qualitative considering these models were designed with a distinct purpose. The THT model focuses on the interpretability of its inner workings that allows generating a metric of pulse clarity over time for symbolic stimuli.

Because our model takes as input a series of onset times, we needed to obtain them from the audio recordings of the dataset. In order to properly compare the procedure in THT with the mentioned models, we used different onset detection algorithms for each case. To compare against BeatRoot [17], we used the onset extraction algorithm provided by the same software. To compare against Böck et al. 2016 [23] we used the onset detection function provided in the *madmom* package. To compare with Böck et al. 2017 [25], we used the same onset detection as with Böck et al. 2016 with the setting to work online (causally) enabled.

The THT model outputs the entire tracking history as a result of the tracking process (see Table 1). From there we must build an actual beat track, a set of designated beat times. We performed this conversion by moving through the rhythmic passage and projecting the highest scoring hypothesis over-time. For each onset event we select the highest scoring hypothesis at that time and project forward from that point until right after a new onset event is encountered. At that time, scores might have changed, so a new top hypothesis is selected, possibly being the same as before. The selected hypothesis is projected and the entire process is repeated until no new onsets are found. Although we are performing this conversion after the tracking was done, it can be done during the tracking process.

**Fig 9:**
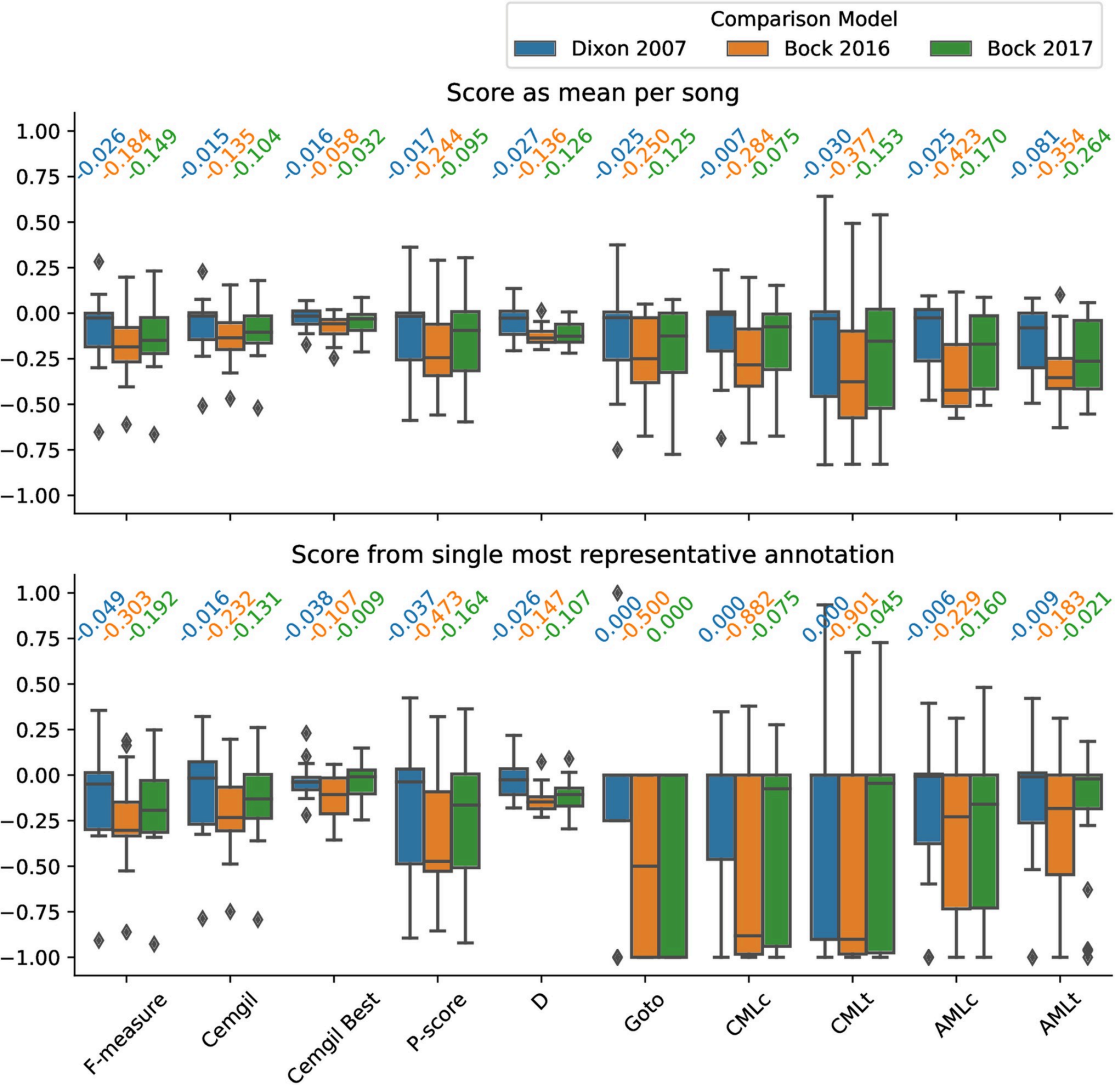
Comparison of THT beat tracking with Dixon 2007 [17], Böck 2016 [23] and Böck 2017 [25]. Score difference is presented as a distribution for each metric in the mir_eval package [38]. Each distribution is gathered from 20 points, one for each excerpt in the dataset. Score differences are calculated in two modalities, as the *mean* across annotators or by selecting a *single* most representative annotator per excerpt. Above each boxplot the median of each distribution is presented in numbers.

The MIREX Audio Beat Tracking competition uses several different evaluation scores with values between 0 and 1 [37]. We will use the implementation provided in the mir_eval python package [38]. Having produced six beat tracks for each excerpt (three from THT with the three onset detection functions and the outputs from BeatRoot, Böck et al. 2016 and Böck et al. 2017) the evaluation metrics from mir_eval were calculated for each beat track against each tapping annotation. In Fig 9, we present the distribution of score differences between THT and the comparison models. Each distribution consists of 20 points where each point is the score difference between THT and the comparison model for one of the excerpts. These differences were calculated in two modalities. In the *mean* modality (top), we calculated the score of THT and the comparison model against each annotator, then took the mean across annotators and finally the difference between the means for each excerpt. In the *single* modality (bottom), a single annotation was used for each excerpt by selecting the most representative tapping annotation—the one that scored best against all other annotations in average using the selected metric in each case. We present the distribution of score differences between THT and the comparison model when comparing their beat tracks with the selected annotation.

For all measures we see the general trend that THT scores closer to BeatRoot, then Böck et al. 2017 and finally Böck et al. 2016. Then, the different metrics provide different information about the beat tracking produced. P-Score, Cemgil and F-measure scores consider the percentage of agreement between two beat sequences by counting the number of times both propose a same instant as a beat time. A time window is used when deciding if two beat times are coinciding. For these metrics, we see the median score difference is less than 0.05 for BeatRoot and 0.2 for Böck et al. 2017. Comparing with Böck et al. 2016, we see the score difference is greater when considering only the most representative annotation than when using the average between annotators. The Cemgil Best and D (Information Gain) metric not only compare the annotations but also allow the beat track to have a different metric level than the annotation, i.e.: half the period and/or counter-phase. These scores show a great decrease in both median and variability of the score difference for all comparisons. This is indicative of THT having a bias towards a different metric level. Upon manually inspecting the trackings, we see a bias towards using half the annotated period. The Goto metric was intended for music with a steady inter-beat interval and assigns each beat tracking versus annotation comparison either a 1 (correct) or a 0 (incorrect). The Continuity metrics (CMLc, CMLt, AMLc, AMLt) add an additional requirement that several contiguous beat times must match a beat annotation consistently. For these metrics, we see the spread of the score difference distribution against the comparison models is greater. This requirement is particularly sensitive if the beat track selected a faster beat period than the annotator. The AMLc and AMLt metrics also perform a resampling of the model’s beat track to account for different metric levels. Although for these metrics the score difference is closer to 0 and less spread than from Goto, CMLc and CMLt; a greater difference than with the first mentioned scores remain. The tracking produced by THT may contain off-phase beat events due to the top hypothesis selection process, hindering the trackings continuity.

Having the distribution of score differences centered close to 0 (median > -15%) in the Cemgil Best and Information Gain (D) metrics shows that the beat track produced by the THT model is musically sensible. This means the beats produced are overall correct in phase and have a musically acceptable period (twice as fast). The bias towards a faster tempo is also reasonable in the context of our objective of modeling pulse clarity. Empirical evidence has shown that when a subdividing pulse is present, tapping variability decreases [39, 40] and pulse saliency is increased [35]. Selecting a more reasonable tactus rate from the highest scoring hypothesis could be done on a secondary step from the output of the THT model, similarly to the scoring procedure.

The bias towards faster tempo and lack of continuity in the beat tracking can be addressed by changing the procedure with which a beat track is obtained from THT’s output. We tested a new procedure that selects a slower tempo and avoids quick changes in the tracking. We present the updated score difference distributions in S1 Fig. S1 and S2 Tables present the median of the score difference distribution for the new procedure with the change in the median value when compared with the previous distribution. With the adaptations, score difference medians are below 0.1 for all metrics when comparing with Dixon 2007 [17] and most metrics when comparing Böck et al. 2017 [25]. During the new procedure, when a new top hypothesis is selected, its period is adjusted to be within tapping speed and the best phase corresponding to the previous period is selected. To adjust the period, it is multiplied by two, if required, to be greater than 375 ms (indicating a beat period smaller than 160 bpm). This limit was obtained from the maximum average tapping speed of the tapping experiment exploring tapping rates from [35]. We also improved continuity by requiring a new hypothesis to be better than the last top hypothesis for more than 3000 ms before it is adopted.

The results also point towards other possible enhancements. More prominently, the score difference in the continuity metrics indicate room for improvement in defining beat positions. Dixon’s model partially solves this because it tries to snap selected beat times to onset times whenever possible. THT, on the other hand, selects all the beat times predictively and onset information is only used after it is heard. Böck et al. models use full acoustic information as input. Our focus on symbolic input restrains this approach. These models are also trained on large datasets allowing them to learn tapping patterns. Including parameters within the model that can be trained with beat tracking datasets must be balanced with the interpretability of the model, considering our objective of modeling pulse clarity. Böck et al. 2016 also uses information of the entire music stimulus to decide beat positions. The score difference distribution closer to zero when comparing with Böck et al. 2017, that is restrained to past information, exhibits this advantage.

## 3 Discussion

The current paper presents THT, a model of beat expectation over time. The model presented analyzes different beat interpretations for a rhythmic passage, estimates the rhythmic congruency of each interpretation and collects the evolution of the interpretations and their congruency over time. The model is based on beat tracking models from the Music Information Retrieval field, performing adaptations that allow inspecting the inner workings of the model while it is exposed to a symbolic rhythmic passage.

We focused on using the model’s output to produce a pulse clarity metric that develops over time as the model ‘listens’ to the rhythmic passage. This metric was contrasted with empirical data from participants tapping the beat to various musical stimuli from the MIREX Beat Tracking training dataset [30]. We used tapping precision of the participants as a proxy for the strength of the pulse induced by the stimuli. The mean pulse clarity of our model presented a negative correlation with tapping variability, indicating that a smaller pulse clarity score relates to more variable tapping. The correlation was comparable with the one obtained from the pulse clarity model most commonly used [6]. The difference between the approaches consists in our model being able to analyze symbolic data and providing a continuous metric. The dynamics of the metric were further assessed on a set of synthetic stimuli that presented situations where pulse clarity and the selected beat interpretation were expected to change mid passage. The model behaved mostly as expected on these examples.

Finally, we evaluated whether the beat tracking produced by the model corresponded to the beat perceived by human listeners. Using again the MIREX dataset, we compared the beat tracking score of the THT model against state-of-the-art models. Results showed the beat track generated by THT was musically sensible but presented a bias towards twice as fast beat time sequences. The bias is consistent with empirical literature on pulse clarity, since rhythms with subdivisions of the main beat more strongly induce the beat [35, 39]. This bias should be corrected or taken into account if THT’s beat track is to be used for further metrical analysis.

The model presented here provides several parameters that relate to the cognitive process of estimating the beat which can be adjusted with data from tapping experiments on carefully designed rhythmic stimuli. The *d* and *m* parameters for the hypothesis correction (see Eq 4) account for the sensitivity to non-beat onsets and the speed with which a beat hypothesis is modified, respectively. Also, the correction process and the congruency score (see Eq 2) can be tuned to weight differently the concurrency of onsets and beats with respect to how long ago they were heard. From the comparison with Böck et al. 2016 [23] and Böck et al. 2017 [25], we observed that informing the model with input richer than onsets and training data improves beat tracking performance. These enhancements should be done with care to preserve the model’s transparency that is useful to analyze rhythmic stimuli.

This work is framed in a larger goal to understand the mechanisms through which music, and particularly rhythm, generates affect. Theories proposed over the last 60 years focus on the role of expectations [1–3, 41, 42]. Music is repetitive and structured, hence allowing to generate predictions on how it will unfold. Yet it also changes its structure delivering pleasurable surprise. Empirical evidence has provided support for the relevance of expectations in musical affect. Sloboda [43] performed a survey to collect listener’s musical experiences that had evoked physical responses such as shivers, laughter or goose bumps. Upon analysis of the musical piece, most cases included situations where expectations were violated. Neuroimaging has also presented evidence of the relevance of expectations in musical pleasure given that dopamine release is increased in anticipation to peak pleasurable experiences [44].

Vuust and Witek [3] framed musical pleasure within the cognitive framework of Predictive Coding. Within this framework, musical affect emerges under unfulfilled musical expectations. This is consistent with the prediction error reward signal seen in the reward system [42]. As a consequence, musical affect is expected to have an inverted-U shape relationship with predictability. High predictability that is fulfilled yields low aesthetic responses because no predictions are defied. Completely unpredictable stimuli is also of low valence since no reasonable expectation emerges. A sweet spot where predictions can be defined but are not always delivered yield the highest pleasure [45].

In rhythms, this relationship between music and pleasure was observed by measuring rhythmic complexity through *syncopation* [10]. In western musical theory, beat times are grouped and subdivided having different structural importance [4]. We expect more salient events on more important beat times [28]. Syncopation is a rhythmic feature describing accents in otherwise unaccented musical locations, being a subversion on rhythmic expectations. On an analysis and review by Thul and Toussaint [11], models of rhythmic complexity that provided measures closer to empirical estimates of complexity were those measuring syncopation. These models of rhythmic complexity require a prior definition of the beat and its hierarchical organization, are intended for short excerpts and expect onsets to be aligned to the beat and its subdivisions. This prevents using them to analyze longer stimuli with imprecise expressive timing as it would be expected in actual performances. They also do not exhibit whether expectations are built and subverted.

The THT model is a first step towards analyzing rhythmic expectations such as syncopation on expressive performances. The model produces estimates of musical subdivisions of the beat which can be used to estimate higher hierarchical groupings (i.e.: the meter). The model also provides a clarity score for the proposed beat that can be used to estimate the confidence on the generated expectations and whether it is relevant if they are unfulfilled. It further allows inspecting if one interpretation is proposed by the rhythm at first and then challenged. For example, the model can be used to design stimuli that transitions from a high to a low congruency score situation considering the listening experience. By having people listen to it and rate how pleasurable they were, we can explore the effect of changes in beat expectations to affect. Another possible design would have rhythmic stimuli that departs and then returns to a high congruency situation. This would allow exploring whether the scenario of relaxation-tension-relaxation found in harmonic musical cadences translates to rhythms. Music is a stimulus that first and foremost develops in time and, as such, should be studied in that context [46].

## 4 Data and software availability

The THT implementation in python used for this work is available at [32]. The repository contains installation and usage instructions. The repository also contains the dataset of 4 synthetic rhythmic examples used in the *Changes in pulse clarity* evaluation subsection.

## Supporting information

S1 Table Score difference median of THT’s adapted beat tracking comparison using all annotations. Each column presents the median for the distribution of score difference versus the defined model. The change in the median value is shown in parenthesis. A negative value indicates the median difference was reduced. Each row presents a different score metric for the beat tracking task.
(PDF)

S2 Table Score difference median of THT’s adapted beat tracking comparison using most representative annotation. Each column presents the median for the distribution of score difference versus the defined model. The change in the median value is shown in parenthesis. A negative value indicates the median difference was reduced. Each row presents a different score metric for the beat tracking task.
(PDF)

S1 Fig Comparison of THT beat tracking with an adapted procedure against Dixon 2007 [17], Böck 2016 [23] and Böck 2017 [25]. Similarly to Fig 9, each boxplot depicts the distribution of score differences between THT and a comparison model are presented. Beat tracking scores were calculated as mean score of all annotations (top) or considering only the most representative annotator (bottom). Median of the difference distribution are presented above each boxplot.
(TIF)

S1 File (ZIP)

S2 File (ZIP)
