# Supplementary material for: From beat tracking to beat expectation: Cognitive-based beat tracking for capturing pulse clarity through time
Source: PLoS One. 2020 Nov 18;15(11):e0242207. doi: 10.1371/journal.pone.0242207 (PMC7673539; doi:10.1371/journal.pone.0242207)
Supplement: S1 Table — Each column presents the median for the distribution of score difference versus the defined model. The change in the median value is shown in parenthesis. A negative value indicates the median difference was reduced. Each row presents a different score metric for the beat tracking task. (PDF) [file pone.0242207.s001.pdf]

S1 Table. Score difference median of THT's adapted beat tracking comparison using all annotations.

Comparison Model<br>Score Type	Bock 2016	Bock 2017	Dixon 2007
F-measure	-0.100 (-0.084)	-0.090 (-0.059)	-0.022 (-0.004)
Cemgil	-0.085 (-0.050)	-0.060 (-0.044)	-0.015 (-0.001)
Cemgil Best	-0.074 (0.016)	-0.094 (0.062)	-0.033 (0.017)
P-score	-0.074 (-0.170)	-0.042 (-0.053)	-0.022 (0.005)
D	-0.043 (-0.093)	-0.019 (-0.108)	0.026 (-0.053)
Goto	-0.262 (0.012)	-0.088 (-0.037)	-0.038 (0.013)
CMLc	-0.126 (-0.157)	-0.023 (-0.052)	-0.000 (-0.007)
CMLt	-0.108 (-0.269)	-0.034 (-0.119)	-0.029 (-0.001)
AMLc	-0.221 (-0.202)	0.000 (-0.170)	0.045 (-0.070)
AMLt	-0.251 (-0.102)	-0.072 (-0.192)	-0.001 (-0.080)

Each column presents the median for the distribution of score difference versus the defined model. The change in the median value is shown in parenthesis. A negative value indicates the median difference was reduced. Each row presents a different score metric for the beat tracking task.
