# Supplementary material for: From beat tracking to beat expectation: Cognitive-based beat tracking for capturing pulse clarity through time
Source: PLoS One. 2020 Nov 18;15(11):e0242207. doi: 10.1371/journal.pone.0242207 (PMC7673539; doi:10.1371/journal.pone.0242207)
Supplement: S2 Table — Each column presents the median for the distribution of score difference versus the defined model. The change in the median value is shown in parenthesis. A negative value indicates the median difference was reduced. Each row presents a different score metric for the beat tracking task. (PDF) [file pone.0242207.s002.pdf]

S2 Table. Score difference median of THT's adapted beat tracking comparison using most representative annotation.

Comparison Model<br>Score Type	Bock 2016	Bock 2017	Dixon 2007
F-measure	-0.173 (-0.130)	-0.112 (-0.081)	-0.025 (-0.024)
Cemgil	-0.105 (-0.127)	-0.062 (-0.069)	-0.003 (-0.014)
Cemgil Best	-0.079 (-0.028)	-0.036 (0.026)	-0.011 (-0.027)
P-score	-0.091 (-0.382)	-0.039 (-0.126)	-0.008 (-0.028)
D	-0.016 (-0.131)	-0.027 (-0.079)	0.026 (-0.052)
Goto	0.000 (-0.500)	0.000 (0.000)	0.000 (0.000)
CMLc	-0.344 (-0.538)	-0.023 (-0.051)	0.000 (0.000)
CMLt	-0.055 (-0.846)	-0.038 (-0.007)	0.000 (0.000)
AMLc	-0.428 (0.199)	-0.165 (0.006)	-0.032 (0.026)
AMLt	-0.147 (-0.036)	-0.032 (0.011)	-0.053 (0.043)

Each column presents the median for the distribution of score difference versus the defined model. The change in the median value is shown in parenthesis. A negative value indicates the median difference was reduced. Each row presents a different score metric for the beat tracking task.
