# Supplementary figures and images for: From beat tracking to beat expectation: Cognitive-based beat tracking for capturing pulse clarity through time
Source: PLoS One. 2020 Nov 18;15(11):e0242207. doi: 10.1371/journal.pone.0242207 (PMC7673539; doi:10.1371/journal.pone.0242207)

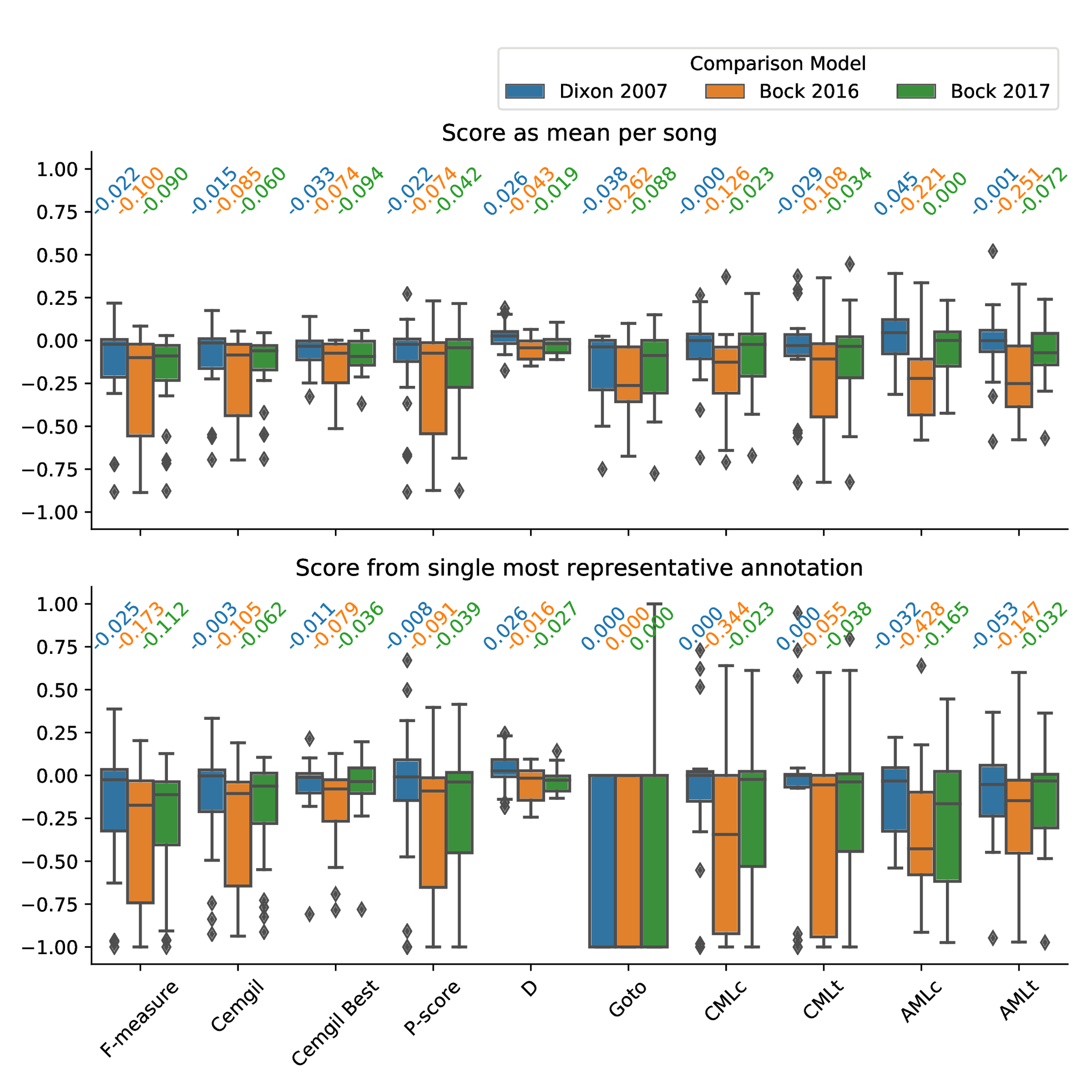

Supplement: S1 Fig — Similarly to Fig 9, each boxplot depicts the distribution of score differences between THT and a comparison model are presented. Beat tracking scores were calculated as mean score of all annotations (top) or considering only the most representative annotator (bottom). Median of the difference distribution are presented above each boxplot. (TIF) [file pone.0242207.s003.tif]
